# Global hydrogen reservoirs in basement and basins

**DOI:** 10.1186/s12932-017-0041-4

**Published:** 2017-03-20

**Authors:** John Parnell, Nigel Blamey

**Affiliations:** 1 School of Geosciences, University of Aberdeen, Aberdeen, AB24 3UE UK; 2 Department of Earth Sciences, Brock University, 500 Glenridge Avenue, St. Catharines, ON L2S 3A1 Canada

**Keywords:** Hydrogen, Granites, Fluid inclusions, Deep biosphere

## Abstract

**Background:**

Hydrogen is known to occur in the groundwaters of some ancient cratons. Where associated gases have been dated, their age extends up to a billion years, and the hydrogen is assumed also to be very old. These observations are interpreted to represent the radiolysis of water and hydration reactions and migration of hydrogen into fracture systems. A hitherto untested implication is that the overwhelming bulk of the ancient low-permeability basement, which is not adjacent to cross-cutting fractures, constitutes a reservoir for hydrogen.

**Results:**

New data obtained from cold crushing to liberate volatiles from fluid inclusions confirm that granites and gneiss of Archean and Palaeoproterozoic (>1600 Ma) age typically contain an order of magnitude greater hydrogen in their entrained fluid than very young (<200 Ma) granites. Sedimentary rocks containing clasts of old basement also include a greater proportion of hydrogen than the young granites.

**Conclusions:**

The data support the case for a global reservoir of hydrogen in both the ancient basement and in the extensive derived sediments. These reservoirs are susceptible to the release of hydrogen through a variety of mechanisms, including deformation, attrition to reduce grain size and diagenetic alteration, thereby contributing to the hydrogen required by chemolithoautotrophs in the deep biosphere.

## Background

Measurements of groundwaters in Precambrian cratons show that they consistently contain hydrogen (data reviewed in [1–3]). The hydrogen is attributed to the long-term radiolysis of water due to natural radioactivity [4] and hydration reactions, including serpentinization and oxidation of ferrous iron [2]. Evidence from noble gas composition shows that gases may have been trapped in the crust for up to a billion years [5–7], and although hydrogen is not dated directly, longevity is implied by the association with dated gases [5], and the genetic link between radiolysis and dated radiogenic helium.

The measurement of hydrogen has focussed on crystalline basement, rather than in sedimentary basins. Yet sedimentary basins are dominated by siliciclastic sediment, that sediment is dominated by the mineral quartz, and most quartz is derived from granites. More generally, siliciclastic sediment ultimately has a basement source, albeit recycled through phases of sedimentary deposition and erosion. An implication of the derivation from crystalline basement is that a signature of hydrogen in the basement could be conferred to the sediment. The ultimate provenance of sediment is evident from the dating of detrital zircon grains. Both modern sand, and sandstone in the geological record, contains a substantial proportion of grains derived from basement sources of early-mid Proterozoic age [8, 9]. This reflects an episode of anomalous crustal growth with globally extensive granite emplacement [10, 11]. We therefore might expect the sediment to carry the heritage of the hydrogen-rich Precambrian basement. A significant proportion of the hydrogen generated by radiolysis in basement rocks, possibly two-thirds [2], is resident in fluid inclusions, and these inclusions survive in the derived sediment particles.

The composition of gases trapped in fluid inclusions can be measured using cold crushing into a mass spectrometer. This technique was developed for the investigation of ore deposits and geothermal systems, and has subsequently found application to diverse crystalline and sedimentary rocks [12–14]. We have used this approach to test:

1. If the hydrogen occurs widely in the entrapped fluid in old basement rocks, as implied by the gas released from Precambrian cratons.
2. If the importance of age can be demonstrated by comparison with data in young basement rocks.
3. If hydrogen also occurs in the derived sediment, as we predict here.

Most water in granitic basement is in post-magmatic secondary fluid inclusions, with only a comparatively minor component originating from the granite melt and forming glassy melt inclusions or rare primary aqueous fluid inclusions [15]. The secondary aqueous fluid inclusions in granites represent hydrothermal fluid from a range of origins, including fractionation of the original melt, convection systems driven by the hot magma, and later fluids focussed on the granite because it has become a structural/topographic high [16, 17]. The entrapped fluid in granitic basement is, therefore, a good record of its post-emplacement history.

## Methods

**Fig. 1:**
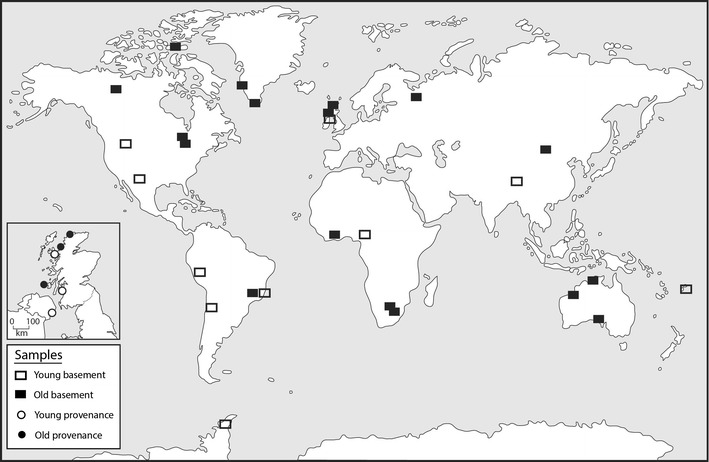
Global map of young and old crystalline basement samples measured for hydrogen content. *Inset* shows map of sediment samples from Scotland and Ireland with provenance from young and old basement

**Fig. 2:**
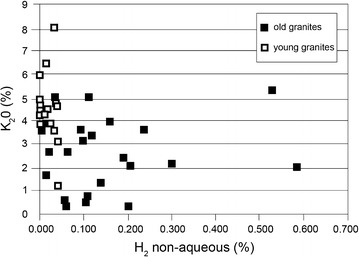
Mean hydrogen contents (% of non-aqueous gas) released by crushes of granitic and other basement rocks of Archean and Palaeoproterozoic age (>1.6 Ga) and young (<200 Ma) age, plotted against potassium content. (Data sources for ages and potassium contents in Table 1)

**Fig. 3:**
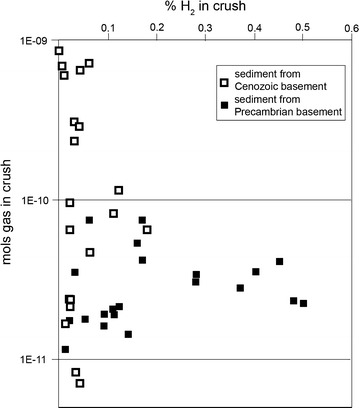
Hydrogen contents (% of total gas) released by individual crushes of quartz particles derived from Cenozoic basement and Palaeoproterozoic basement, northwest Britain (sample locations in Fig. 1 *inset*). Greater hydrogen contents were measured from particles from older basement

We have tested the occurrence of hydrogen in Precambrian basement (Fig. 1) by measuring the composition of gases released by cold crushing of granitic/gneissose basement samples. We have also analysed a set of young granites, to test if age is a critical factor in gas composition. 25 samples older than 1600 Ma, and 17 samples younger than 200 Ma were analysed (Fig. 2). Both old and young samples have a global distribution (Fig. 1). Where possible, the samples are granitoids. Some of the older samples are gneisses or conglomerates, which should have broadly comparable mineralogy and chemistry. Samples containing high concentrations of uranium were excluded, with the exception of the Moeda Formation conglomerate from Brazil. Further, we have analysed quartzose sediment derived from old and young basement, to test if gas signatures are preserved in clasts derived from erosion of basement. These analyses were restricted to unconsolidated sediment, to allow isolation of the individual clasts and avoid any contribution from mineral cements. A comparison was made using sediment derived from three Cenozoic and three Palaeoproterozoic granitic basement sources within a small area of crust in northwest Britain (Fig. 3). Quartz particles in sediment derived from Cenozoic granites in the Mourne Mountains (Northern Ireland), Arran and Skye (Scotland) were compared with quartz particles in sediment derived from Proterozoic granites/gneisses in Islay, North Uist and Sutherlandshire (Scotland).

The cold crush method involves analysis by mass spectrometry conducted in high vacuum as described in [12–14]. Each session was preceded and followed by analysis of one microlitre capillary tubes for calibration. Atmosphere was also introduced to verify the calibration using 100–200 acquisitions for both the sample and atmosphere standard. A match head sized sample (about 250 microns) is crushed incrementally under a vacuum of ~10^−8^ Torr, producing 6–10 successive bursts, which remained in the vacuum chamber for 8–10 analyser scans (~2 s) before removal by the vacuum pump. This method does not require a carrier gas and volatiles are not separated from each other but released simultaneously into the chamber. The act of incremental crushing may open a single inclusion or multiple fluid inclusions. The data acquisition is performed by means of two Pfeiffer Prisma quadrupole mass spectrometers operating in fast-scan, peak-hopping mode. Routinely the system analyzes for the following gaseous species including H_2_, He, CH_4_, H_2_O, N_2_, O_2_, Ar, and CO_2_. The volatiles are reported in mol%. The instrument is calibrated using Scott Gas Mini-mix gas mixtures (with 2% uncertainty), capillary tubes filled with gas mixtures (with 1% uncertainty), and three in-house fluid inclusion gas standards. The amount of each species is calculated by matrix multiplication [18] to provide a quantitative analysis. The 2-sigma detection limit for most inorganic species is about 0.2 ppm for aqueous fluid inclusions. Instrumental blanks were also analyzed routinely to assess if gases were produced during the crushing process. The mass spectra remained at background during crushing of blanks indicating that gases released are not sourced from the crushers or hardware. Linearity of the mass spectrometer was confirmed up to nitrogen partial pressures of 10^−6^ Torr, which is orders of magnitude higher than routine operating pressures. The error across the linear range of the mass spectrometer is estimated from the standard deviation for capillary tube measurements of the N_2_/Ar ratio. This covers the noise and the area under the peak curve. The measurements indicate a maximum error of 1%. Precision and accuracy vary between species. The amount of each species was calculated by matrix methods to provide a quantitative analysis, which is corrected for the instrumental background. Nine capillary tubes with encapsulated atmosphere were analyzed and yielded N_2_/Ar ratios of 83.2 with a standard deviation of 1.4, within error of the atmospheric N_2_/Ar ratio of 83.6. This translates into 0.5% accuracy for artificial inclusions made under laboratory conditions. Precision using natural inclusions for the major gas species measured is generally 2–5%, these being dependent on summed errors derived from instrument noise, linearity of the mass spectrometer, uncertainty of standards, blanks, interferences, and measurement of sensitivity factors. Before analysis, the crushing area and the bellows of the crusher were cleaned using potassium hydroxide. The apparatus is also routinely cleaned with isopropanol. Thereafter, the crushing chamber is baked at about 150–200 °C for 72 h before loading and analysing the samples at room temperature the next day. The crushing area is isolated from the main chamber so that the main chamber can be baked out every evening.

The hydrogen data is converted to the proportion of the non-aqueous component, to avoid misleading inferences from variations in the abundance of aqueous fluid inclusions, which dominate the entrained fluid.

### Data

The data for the Precambrian basement samples (Table 1; Fig. 2) shows that consistently there is a hydrogen component to the entrained fluids. The mean value for the measurements is 0.142% non-aqueous gas. By contrast, the data for the younger basement samples are consistently in the range from 0 to 0.04%, with a median value of 0.019%, A single sample from the younger set, from Chile, yielded a high value of 0.13%: however, this sample was subsequently found to be from rocks being exploited for uranium, and so is excluded from the comparison. The total data set shows a marked difference between the older and younger basement samples, with an order of magnitude more hydrogen in the older samples. The modern sediment in northwest Britain yielded low hydrogen contents from the samples derived from Cenozoic sources, but higher hydrogen contents from some of the samples derived from Precambrian sources (Fig. 3).

**Table 1:** Contents of H_2_, He, and mols of gas measured in samples of old and young basement, and sediments with old and young provenance

Sample code/location	Rock type	H_2_ (%)	He (%)	H_2_O (%)	H_2_ (% non-water)	Mols gas	K_2_O (%)	Age	References
*Old basement*									
JP210 Devon Is	Granite	0.46	0.000	83.27	0.027	1.4 * −09	nd	~1.9 Ga	[36]
JP212 White Sea, Karelia	Granite	0.04	0.000	93.23	0.006	6.1 * −10	3.6	~2.8 Ga	[37, 38]
JP219 Chiobino, Karelia	Granite	3.11	0.001	86.80	0.236	6.4 * −11	3.6	~2.8 Ga	[37, 38]
JP200 Ceannabeinne	Gneiss	1.67	0.000	97.14	0.584	3.3 * −10	2.0	~3.1 Ga	[39]
JP201 South Harris gneiss	Gneiss	0.82	0.005	92.17	0.105	1.9 * −10	0.6	1.88 Ga	[39]
JP147 Creighton, Sudbury	Granite	2.03	0.005	96.17	0.530	5.4 * −11	5.3	2.33 Ga	[40, 41]
JP177 North Uist gneiss	Gneiss	0.61	0.000	90.40	0.064	3.1 * −11	2.7	~3.1 Ga	[39]
JP92 Devon Is	Granite	0.67	0.000	84.67	0.044	2.5 * −09	nd	~1.9 Ga	[36]
JP169 Barra	Gneiss	0.17	0.000	92.49	0.023	5.7 * −10	2.7	~3.1 Ga	[39]
JP139 Moeda Fm	Conglomerate	0.77	1.660	86.50	0.057	5.5 * −10	0.6	~2.5 Ga	[42]
8852 Tarkwa	Conglomerate	0.33		80.20	0.017	3.1 * −10	1.7	2.06 Ga	[43, 44]
9293 Shaw batholith (Aus)	Gneiss	0.44	0.001	97.86	0.206	9.3 * −11	2.1	3.45 Ga	[45]
9299 Mt Edgar batholith (Aus)	Gneiss	0.10	0.001	99.47	0.189	8.3 * −11	2.4	3.3 Ga	[46]
9298 Swaziland Ngwane gneiss	Gneiss	0.25	0.012	97.87	0.117	1.2 * −10	3.4	~3.5 Ga	[47]
9300 MSC105 Acasta TTG	Gneiss	0.53	0.001	97.39	0.203	1.7 * −10	0.4	~4.0 Ga	[48]
9301 MSC110 Acasta TTG	Gneiss	0.15	0.000	97.44	0.059	3.0 * −10	0.4	3.96 Ga	[49]
JP221 Gawler Craton (Aus)	Gneiss	0.20	0.014	98.00	0.100	2.0 * −10	3.2	~2.5 Ga	[50]
JP224 Cullen (Aus)	Granite	0.34	0.001	90.03	0.034	3.0 * −10	5.0	1.76 Ga	[51]
JP233 Bass Lake Ontario	Granite	0.10	0.000	97.78	0.045	1.8 * −10	nd	~2.7 Ga	[52]
KV3 Kaap Valley pluton SA	Tonalite	0.78	0.003	94.46	0.141	8.4 * −11	1.3	3.2 Ga	[53]
JP236 Rhinns Complex	Syenite	0.84	0.000	92.41	0.111	1.1 * −10	5.0	1.78 Ga	[54, 55]
JP246 Nuuk, Greenland	Granite–gneiss	2.72	0.004	74.92	0.108	1.1 * −10	0.8	~3.05 Ga	[56]
JP249 Gairloch Pier	Granite	0.54	0.001	98.20	0.300	2.4 * −10	2.2	1.9 Ga	[57, 58]
JP262 Nanortalik, Greenland	Gneiss	1.12	0.070	92.91	0.158	1.4 * −10	4.0	~1.8 Ga	[59, 60]
KCL19 Liangchen, N China	Granulite	1.29	0.001	86.36	0.095	7.1 * −11	3.62	~2.5 Ga	[61]
*Young basement*									
JP205 Tibet	Granite	0.00	0.000	94.38	0.000	7.1 * −10	4.3	~50 Ma	[62]
JP206 Tibet	Granite	0.02	0.000	96.51	0.006	2.9 * −10	4.3	~50 Ma	[62]
JP207 Antarctic	Granodiorite	0.14	0.000	96.59	0.041	1.5 * −10	3.1	167 Ma	[63, 64]
JP208 Antarctic	Granodiorite	0.08	0.000	97.98	0.040	1.4 * −10	4.7	167 Ma	[63, 64]
JP209 Antarctic	Granodiorite	0.15	0.001	93.50	0.023	3.7 * −10	3.9	167 Ma	[63, 64]
JP214 Nigeria	Granite	0.00	0.000	81.85	0.000	2.0 * −09	4.5	~160 Ma	[65]
JP215 Nigeria	Granite	0.02	0.000	62.06	0.001	6.8 * −09	4.7	~160 Ma	[65]
JP216 Fiji	Granite	0.04	0.000	99.06	0.043	1.1 * −10	1.2	~15 Ma	[66]
JP218 Ireland	Granite	0.10	0.000	97.34	0.038	3.1 * −10	4.8	56 Ma	[67, 68]
9292 Chuqui, Chile	Granodiorite	0.01	0.000	95.37	0.002	5.1 * −10	~6.0	36 Ma	[69, 70]
9305 New Mexico Sugar Loaf	Grandiorite	0.01	0.000	98.51	0.007	6.7 * −11	3.9	35 Ma	[71]
JP223 Arran	Granite	0.00	0.000	99.13	0.000	1.3 * −10	4.9	60 Ma	[67, 72]
JP232 Brazil	Syenite	0.02	0.000	99.41	0.034	4.9 * −11	8.0	79 Ma	[73]
JP234 Peru	Granodiorite	0.10	0.000	96.68	0.030	8.2 * −11	3.6	~10 Ma	[74]
JP229 Skye	Granite	0.03	0.000	98.37	0.018	3.0 * −10	4.5	59 Ma	[72, 75]
JP238 Montana	Syenite	0.06	0.001	96.30	0.016	1.1 * −10	6.5	~52 Ma	[76]
NB New Mexico Questa	Granite	0.07	0.000	95.93	0.017	1.7 * −10	3.9	19 Ma	[77]
9291 El Abra Chile	Granodiorite (U minztn)	0.26	0.000	97.95	0.127	2.3 * −10		37 Ma	[78, 79]
*Sediments with old provenance*									
JP247 Gairloch Pier		0.54	0	96.92	0.175	2.6 * −10			
JP243A Portnahaven		0.13	0	97.28	0.048	1.6 * −10			
JP248 Laxford		0.15	0	96.95	0.049	2.4 * −10			
*Sediments with young provenance*									
JP242A Red Cuillin, Skye		0.02	0	98.77	0.016	5.8 * −11			
JP244 Bloody Bridge		0.07	0	98.68	0.053	3.9 * −10			
JP245 Glen Sannox		0.027	0	91.02	0.003	4.4 * −09			

K_2_O content and ages from literature

## Discussion

The database confirms previous theory [1, 2] that there is a global reservoir of hydrogen in crystalline basement and clarifies that it is resident particularly in old basement. The greater concentrations of hydrogen in the older rocks can be explained by the greater accumulated radioactivity, and hence radiolysis. A major proportion of the radioactivity is from potassium. In a coarse-grained rock like granite, beta-irradiation from potassium is more likely to penetrate beyond grain boundaries into intergranular fluid than shorter-range alpha irradiation from uranium, and potassium is also more pervasively distributed than the uranium in granite, so contributes more widely to radiolysis. Potassium is as abundant in the young basement as it is in the Precambrian basement (Fig. 2), showing that it is age rather than composition that is the control on hydrogen content. The hydrogen contents measured in modern sediments show that the gas signature of the basement rocks is conferred to the derived clasts. The more hydrogen-rich Precambrian basement is reflected in greater hydrogen contents in sediment derived from Precambrian basement than in sediment derived from Cenozoic basement. There are other mineral alteration mechanisms by which hydrogen can be generated in sediments on relatively short time scales (e.g. [19]), but the correlation of sediment provenance with hydrogen content shows that radiolysis is a major contributor.

The hydrogen entrained in basement and derived sediment is available for release by a variety of mechanisms, including solid state diffusion, strain deformation, fault movement and a range of surface erosion processes such as glacial grinding. Many of these mechanisms would involve decrepitation of the fluid inclusions, as is widely observed [20]. The availability of hydrogen in the subsurface in particular is important as a potential fuel for a deep biosphere [3, 21, 22]. Hydrogen may be the predominant source of energy for microbial activity in the subsurface, with a record back to the earliest life on Earth [23].

The signatures in old sediments will be a mixture of hydrogen generated in the provenance basement, and hydrogen generated by radiolysis since sediment deposition. As long as there is still a source of radioactivity, including potassium, radiolysis will continue, especially in fine-grained sediments where a greater proportion of the shorter range alpha irradiation may interact with pore waters [24]. This is conspicuously evident in the Oklo uranium deposits, Gabon, where fluid inclusions in sandstone contain discrete oxygen and hydrogen generated by radiolysis [25, 26]. Where basement and derived sediment must differ in hydrogen generation is in the case of mineralogically mature sands consisting almost exclusively of quartz, where the potassium-bearing phases (especially feldspars) have been eliminated and the potassium becomes concentrated in clay/silt-sized minerals that are deposited under different hydrodynamic conditions. However the quartz may still retain hydrogen generated from adjacent mineral phases before basement and sediment erosion.

The availability of hydrogen in sedimentary basins may be as relevant, if not more so, to supporting a subsurface biosphere, than the availability of hydrogen in the parent basement. In contrast to basement rocks, in which deformation is episodic and spatially focussed, deformation in compacting basins proceeds relatively continuously and widely. Microfractures develop in compacting sands, even before they are fully lithified, and are rapidly re-healed as micron-scale planes containing entrapped fluid, extensively evident in cathodoluminescence images [27, 28]. However, there is evidence for low level availability of hydrogen in shallow sediment, where it is oxidized by microbes, but it is assumed that the hydrogen they process is derived from overlying atmosphere [29, 30]. Microbes also utilize hydrogen where it is available from deep sediment [31] and subsurface crystalline sources, especially through interaction of Fe(II) and water [4, 32–34]. These communities show that where hydrogen is available it is likely to be utilized and this will include hydrogen released from reservoirs in sedimentary rocks.

There is an implication for other rocky planets, which may similarly contain a subsurface reservoir of hydrogen derived from radiolysis, and thereby could support subsurface life. Notably, such life would not require surface water, and so would not be constrained by the ‘Goldilocks Zone’ commonly used to define the limits of habitability [35].

## Conclusions

The measurement of entrained gases using the cold crush method show that hydrogen occurs in both basement and sediments. In detail:

1. Precambrian basement consistently contains entrained hydrogen, at levels an order of magnitude greater than in young (<200 Ma) basement.
2. Modern sediment derived from old and young basement retains the signature of more or less hydrogen, respectively.
3. The high proportion of particles of early-mid Proterozoic age in modern sediments implies that relatively high levels of entrained hydrogen are held in much of that sediment.

These data show that reservoirs of hydrogen occur in both basement and sediment, available to support subsurface microbial activity.
